# Population characteristics and geographic coverage of primary care facilities

**DOI:** 10.1186/s12913-018-3221-8

**Published:** 2018-06-01

**Authors:** Byron Graham

**Affiliations:** Queen’s Management School, Queen’s University Belfast, Riddel Hall, 185 Stranmillis Road, Belfast, BT9 5EE UK

**Keywords:** Accessibility, Facility location, Primary care location, Coverage, General practice, Healthcare need

## Abstract

**Background:**

The location of General Practitioner (GP) facilities is an important aspect in the design of healthcare systems to ensure they are accessible by populations with healthcare needs. A key consideration in the facility location decision involves matching the population need for the services with the supply of healthcare resources. The literature points to several factors which may be important in the decision making process, such as deprivation, transportation, rurality, and population age.

**Methods:**

This study uses two approaches to examine the factors associated with GP accessibility in Northern Ireland. The first uses multinomial regression to examine the factors associated with GP coverage, measured as the proportion of people who live within 1.5 km road network distance from the nearest GP practice. The second focuses on the factors associated with the average travel distance to the nearest GP practice, again measured using network distance. The empirical research is carried out using population and geospatial data from Northern Ireland, across 890 Super Output Areas and 343 GP practices.

**Results:**

In 19% of Super Output Areas, all of the population live within 1.5 km of a GP practice, whilst in 24% none of the population live within 1.5 km. The regression results show that there are higher levels of population coverage in more deprived areas, smaller areas, and areas that have more elderly populations. Similarly, the average travel distance is related to deprivation, population age, and area size.

**Conclusions:**

The results indicate that GP practices are located in areas with higher levels of service need, but also that care needs to be taken to ensure rural populations have sufficient access to services, whether delivered through GP practices or through alternative services where GP practices are less accessible. The methodology and results should be considered by policy makers and healthcare managers when making decisions about GP facility location and service provision.

## Background

Primary care plays an important role in improving the health of surrounding populations [1–3]. However, GP practices must be accessible to facilitate utilisation and better health outcomes [4–7], thus highlighting the importance of considering accessibility in the design and management of healthcare systems. An important component of accessibility is related to geography, and in particular to the distance that must be travelled to reach the facility, which can impact on service utilisation for both preventative and curative interventions [3, 4, 8]. Facility location is also important in maximising cost and utilisation efficiencies in the healthcare system [9], which is crucial in a climate of public funding constraints and for the business efficiency of GP practices. Ensuring equity of access to facilities across the population is also important, and can be particularly challenging in areas with a rural geography, deprivation inequalities, and other social, religious and political divides.

This study focuses specifically on the location of GP practices, which are crucial components of the healthcare system in providing both primary care and functioning as an access point to other parts of the healthcare system. In addition, GP’s provide a range of other services such as vaccinations and smoking cessation [10]. Although the importance of primary care accessibility is recognised, the inverse care law proposes that areas which are most in need of healthcare tend to have poorer levels of access, particularly where market forces are at play [11], although the empirical evidence for this proposition is mixed [12–14].

Deciding on the location of primary care facilities is complex, with accessibility and utilisation influenced by factors in addition to geographical proximity, such as wait time, affordability, healthcare knowledge, and the availability of transportation [4, 15]. When studying access to services it is important to take into consideration the factors associated with population need for the service, such as age, gender, deprivation, and measures of health [15]. These are considered in both the academic literature [10, 13], and by healthcare policy and planning authorities, through for example, capitation formulas [16, 17], which are used in the allocation of funding based on the population need. The ability to access the service should also be considered, along with the need for the service and any predisposing risk factors [15]. Moreover, these factors may also be important determinants of the service mix required by the population. Healthcare decision makers and practice managers should therefore consider these wider factors when making locational decisions and decisions around the services provided, such as opening hours and specialist services.

The accessibility of healthcare facilities can take into account both spatial and non-spatial factors, and can be measured and interpreted in a range of ways. These can include the distance between the population and facility, facilities per population of geographical region, the number of staff and waiting times, satisfaction, utilisation, cost of access, transportation links, catchment areas or coverage, and gravity models [3, 15, 18, 19]. Studies such as Hawthorne [20] have added to these accessibility measures by creating a measure of access based on distance and satisfaction with the service, to take account of the potential for people to travel further to access higher quality care.

Despite the breadth and depth of research into the accessibility of healthcare facilities, challenges remain in both the scientific literature and in healthcare location policy and decision-making. One challenge is that accessibility is a multifaceted concept, with no consistent definition of ‘poor access’ to healthcare services [21]. Moreover, the literature highlights a wide range of predisposing, enabling and need factors important in determining healthcare utilisation [15, 22]. This points towards the complexity of the facility location problem, and makes it challenging to objectively evaluate and plan service provision.

The multifaceted nature of accessibility makes the facility location decision particularly challenging due to the need to take into consideration a range of factors, such as cost effectiveness, the location of existing facilities, planned developments, and government and administrative policies. Despite this, much of the literature on the location of healthcare facility location only includes a small subset of the possible factors influencing the location decision, or focuses only on achieving maximal coverage regardless of the population characteristics influencing the service need.

This study draws on the existing literature on healthcare need, accessibility, and utilisation to examine the factors associated with geographical location of GP practices. Specifically, this study will seek to answer the following research question:

What factors are associated with the location of primary healthcare facilities?

To answer this question, multivariable regression models are developed to examine the factors related to the proportion of each Super Output Area (SOA) residing within 1.5 km of the nearest GP practice, and the average travel distance for each SOA. In contrast to some previous studies which have used Euclidian distance [10, 23], this study uses the more accurate network distance. These measures are combined with other sources of area level data which may be related to the location of the GP practice, such as multiple deprivation, health deprivation, population age, area, and religion. This facilitates a detailed examination of the relationships between accessibility and population characteristics.

This study makes several theoretical and practical contributions to the literature. The first is the use of a novel dataset to study the factors related to the location of healthcare facilities. The paper adds to our understanding of the range of factors that could shape the location of GP practices, providing policy makers with insight about current levels of provision and the factors related to accessibility. In particular, this study will contribute to our knowledge of the population characteristics in which GP practices are located in Northern Ireland. This remains an important topic, with previous studies reporting conflicting findings around the existence of an inverse care law, with some studies finding evidence in support of an inverse care law [12, 13, 24], and others not [10, 25]. However, in general distance based studies tend to show that GP practices are more accessible in more deprived areas [10]. Moreover, the relationship between deprivation and accessibility is made less clear due to differences in the age profile of areas, with more deprived areas consisting of more younger people, compared with less deprived areas consisting of more older people and consequently more age related healthcare needs [26]. This study makes a further contribution to this debate, by considering both deprivation and age.

From a practical perspective, this study will contribute to planning and policy decisions on the location of primary healthcare facilities through the measurement of facility coverage, and its associated factors. The results presented in this study will also help policy makers to examine the extent to which GP practice location is matched to population characteristics.

Identification of areas where accessibility is low is also important to healthcare decision makers in planning the provision of services. This is of current policy relevance in primary care as pharmacies take on some of the roles historically reserved for GP’s such as minor ailment schemes [27], and GP practices expand to take on additional roles [28]. Incorporation of the insights gained in this study could therefore add to the planning and policy discussions around the provision of wider healthcare services. As this study is based on the analysis of publicly available data, it also demonstrates the usefulness of open data in studying healthcare geography and highlights the importance of recent government open data initiatives.

## Methods

### Study setting

This study focuses on the location of GP practices in Northern Ireland. In Northern Ireland there are 343 GP practices and 1710 GP’s serving a population of over 1.9 million registered patients [29, 30]. GP practices are run as small businesses, with practices responsible for day to day management and recruitment of staff [30]. Practices are funded by the Northern Ireland Health and Social Care Board under the current GP contract, but remain as separate businesses [30]. The initial location decision is therefore likely to be crucial, as it is likely to be easier to expand an existing practice by employing additional staff than it would be to open an entirely new facility. There may also be scenarios where alternative services are more appropriate.

The locational decision of GP practices in Northern Ireland is based on criteria that aims to ensure accessibility, taking into consideration factors such as demand, travel distance, new housing developments and difficulties registering on existing GP lists. Equality of access to public services is a particularly important issue in Northern Ireland due to community divides on political and religious grounds. Ensuring equality of provision of public services is also written into Northern Ireland legislation [31], with Section 75 of the Northern Ireland Act 1998 requiring public services to consider equality of provision across the areas of religion, political opinion, race, age, gender, marital status, sexual orientation, disability, and dependants [32]. Equality of access to healthcare services in general has been widely studied in the literature across various geographical areas [33].

### Dependent variables

There is no universally agreed measure of coverage or accessibility in the literature, with studies adopting a variety of measures such as straight line distance [10], drive time [13], availability of bus services [13], floating catchment areas [18, 34, 35], and population to GP ratios. For example, Todd et al. [10] measure the Euclidian distance between population centroids and the nearest GP practice. Rosero-Bixby [23] also uses a distance measure, but also creates an additional weighted index measure taking account other relevant factors.

As the focus of this study is on the factors related to distance accessibility, two measures of geographic accessibility were developed for use as dependent variables. The first measures practice coverage as the proportion of people in each SOA that live within 1.5 km of a GP practice. The second measure of accessibility is the average distance between the population of each SOA and the nearest GP practice.

Both measures were calculated based on network distance using road network data from Ordinance Survey Northern Ireland [36]. Network distance and subsequent mapping was carried out using ArcGIS (version 10.5). The 2017 GP list [37] and the 2017 central postcode directory were used to identify the postcodes and latitude and longitude of the facilities and postcodes in Northern Ireland. The postcode location of the GP practice was identified by combining the GP postcode with the central postcode lookup, which contains the latitude and longitude of every postcode in the United Kingdom. Postcodes have been widely used to calculate distance in previous GIS studies e.g. [10, 38]. On average there are 31 people living at each postcode in Northern Ireland, and there are over 58,000 postcodes. Distance was measured by calculating the network distance between the GP postcode and every other postcode in Northern Ireland.

The proportion of people within the coverage area was calculated by dividing the sum of the usually resident population at each postcode location within 1.5 km of a GP by the total population of the SOA, resulting in a value between 0 and 1. This measure acts as a proxy for individual level travel distance to the nearest GP practice. Due to a high proportion of areas having either full or no coverage, a new variable was computed for use in the subsequent modelling, consisting of four categories: no coverage, low coverage, high coverage, and full coverage. This variable was based on percentage of the population within 1.5 km of a GP practice, with no coverage representing 0%, low coverage greater than 0% and less than 50%, high coverage between 50% and less than 100%, and full coverage indicating 100% of the area is within 1.5 km of a GP practice.

Similar to Todd et al. [10] who used a distance of 1.6 km, a distance of 1.5 km coverage was chosen as representing around a 20 min walk from the persons house to the GP practice. Sensitivity analysis was also carried out based on other distances, as discussed further in the analysis section. The average travel distance to the nearest GP practice was also based on the network distance calculated for the coverage proportion measures. This was calculated by deriving the mean network travel distance for the population of each SOA, again based on the usually resident postcode population and the distance to their nearest GP practice.

### Independent variables

In addition to geographic measures of accessibility, previous studies have considered a range of other factors which may be important in the location of GP practices. These include access to transportation, healthcare needs, rurality, deprivation, and demography [10, 13, 14, 26, 38, 39]. Drawing on the wider literature, the independent variables included in this study were the Northern Ireland Measure of Multiple Deprivation (MDM), health deprivation, the SOA level population aged 0–15 and over 65, the proportion of the area who report being protestant religion, and the size of the area. In total six publicly available datasets were combined to form the final dataset. The 2017 MDM was used in the final models, which is a composite measure made up of 8 sub domains, which provides a deprivation ranking to each area [40]. The health deprivation domain was also included individually due to the particular relevance of this domain in the location of GP practices.

The 2016 mid year population age groups [41] were used as the measure of age. Earlier models included four age bands: 0–15, 16–39, 40–64 and 65+, but the inclusion of all four bands introduced high levels of multicolinearity into the model, probably because of children aged 0–15 cohabiting with their parents or guardians aged 15–64. The final models therefore only include the age bands of 0–15 and 65+. The SOA area was measured in square kilometres as presented in the most recent Northern Ireland SOA’s [42].

Religion was captured in the models using the 2011 census measurement of the percentage of people reporting their religion or religion brought up in was Protestant and other Christian [43]. In the 2011 census, 93.5% of the population selected either this option, or Catholic, with other religions accounting for less than 1% of the population, and no religion accounting for 5.6% of the population. Because of the very small proportions outside of Protestant and Catholic, the models focus on the proportion of the population reporting as Protestant and Other Christian. Car ownership and rurality were included in earlier models, but are not included in the final models as these measures are likely to be captured by the multiple deprivation measure, and SOA area measures respectively.

The final set of variables included in the analysis was determined by the literature reviewed, the data available, and significance in the models. All data included in the study was measured at the SOA level in Northern Ireland, which is the lowest level at which most of the statistical measures of interest are made available. Northern Ireland is broken down into 890 SOAs, with an average of 2000 people or 700 households in each area [44].

### Data analysis

The initial analysis of the dataset involved producing descriptive statistics and visualisations, and investigating the correlation between variables. Due to the ordered nature of the coverage dependent variable ordinal logistic regression would be the best choice as it utilises the information contained in the order of the variables. However, the test of parallel lines assumption was violated. Multinomial logistic regression was therefore used to examine the relationships between coverage and the population characteristics. Ordinary least squares regression was used to examine the relationships between population characteristics and the average travel distance within each SOA. Separate models were built to include multiple deprivation and health deprivation due to high levels of correlation between these two variables.

Sensitivity analysis was carried out by rerunning the multinomial regression models based on different network distances of 750 m, 1 km, 1.25 km, 1.75 km and 2 km. Each model was examined for differences in the significance of the variables compared to the focal 1.5 km model. All data pre-processing, variable calculation, visualisations and initial statistical analyses were undertaken using the R statistical programming language [45], with multinomial regression analysis undertaken in SPSS Version 22.

## Results

**Fig. 1:**
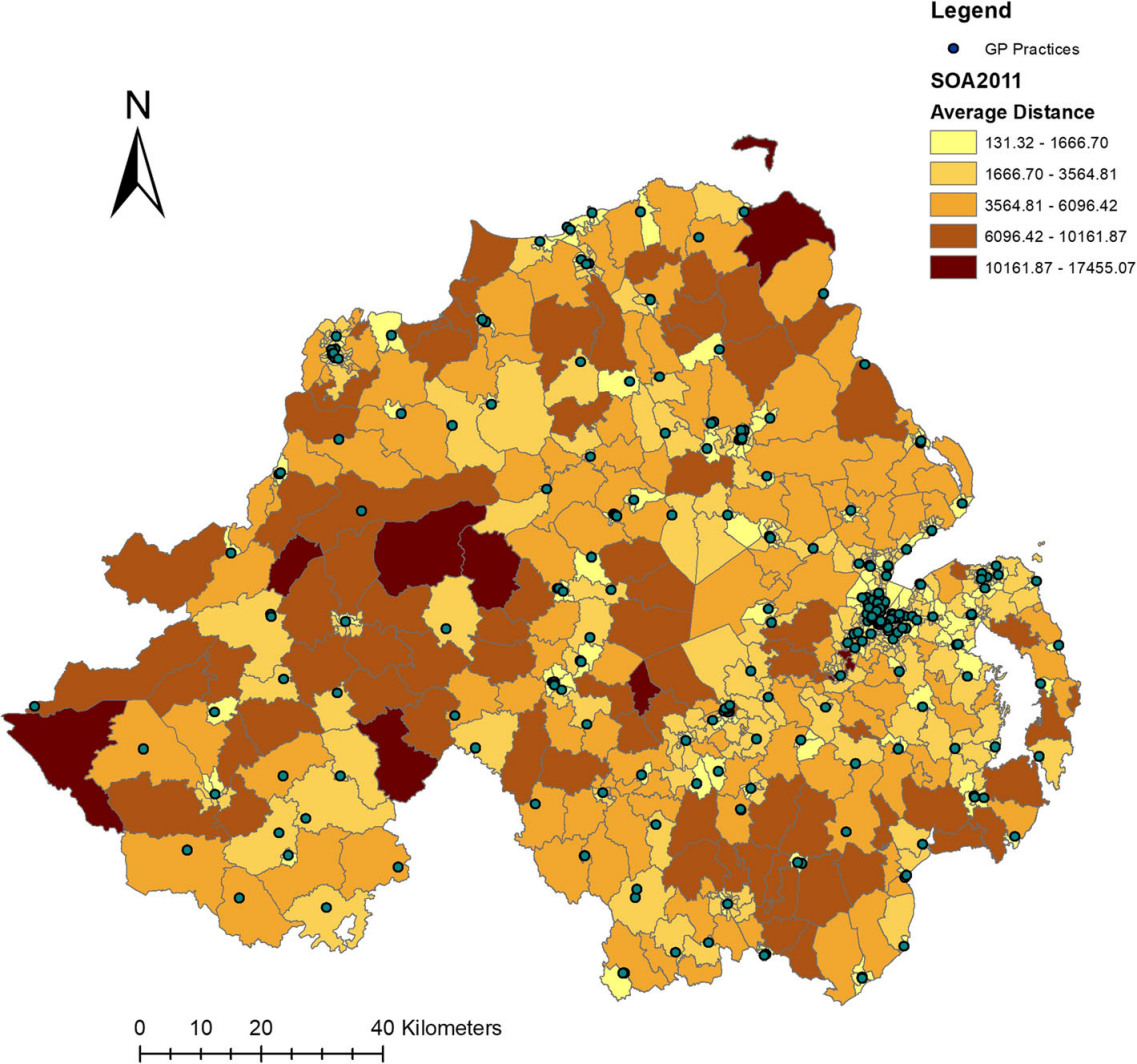
Mean travel distance to the nearest GP practice, and the location of GP practices in Northern Ireland (source: Authors’ own work)

Table 1 summarises the dependent variable and the Northern Ireland SOA level measures that are included in the study. Across all SOA’s the mean network distance between the population and the nearest GP practice is 2684.51 m. This is displayed visually in Fig. 1, which shows a map of Northern Ireland with the mean travel distance within each SOA, along with the location of each GP practice. In 18.7% of SOA’s all of the population live within 1.5 km of a GP practice, and in 24.4% of SOA’s none of the population live within 1.5 km of a GP practice. This highlights the differences in GP coverage levels across the region. SOA’s have a mean size of 15.882 km^2^. Across all SOA’s, a mean of 436 people are aged 0–15, 654 are aged 16–39, 668 are aged 40–64, and 335 are aged 65 and over. The mean MDM and health deprivation rank is 445.5.

**Table 1:** Descriptive statistics

Statistic	N	Mean / (%)	SD	Min	Max
Average Distance to GP	890	2684.51	2561.73	132.93	17,455.07
MDM Rank	890	445.50	257.065	1	890
Health Deprivation Rank	890	445.50	257.065	1	890
Area (km^2^)	890	15.882	29.54	0.11	205.11
Persons Aged 0–15	890	435.95	176.96	48	1663
Persons Aged 16–39	890	654.12	250.942	94	2362
Persons Aged 40–64	890	667.68	200.58	110	1575
Persons Aged 65+	890	334.55	123.57	6	1066
Protestant or Other Christian (%)	890	48.96	29.14	1.5	91.26
Full Coverage (%)	167	18.7			
High Coverage (%)	260	29.2			
Low Coverage (%)	246	27.7			
No Coverage (%)	217	24.4

For subsequent multinomial models the proportion of people in each SOA living within 1.5 km of a GP practice was converted to four levels, ranging from no coverage to full coverage. Table 2 presents the descriptive statistics broken down by the four levels of coverage. The results show that the most deprived areas tend to have the highest level of coverage, across both MDM and health deprivation ranks. Areas with lower coverage also tend to be much larger in terms of geographical area. No consistent pattern seems to emerge from the descriptive statistics around the population age and religion.

**Table 2:** Descriptive Statistics by Coverage Level. Mean with standard deviation in parentheses

	Full	High	Low	None
Average Distance	615.24 (252.14)	1495.02 (1782.43)	3205.39 (2076.71)	5111.68 (2649.21)
MDM Rank	355.64 (295.17)	431.36 (259.25)	494.38 (244.02)	476.18 (216.19)
Health Deprivation Rank	306.57 (256.47)	401.12 (245.67)	529.39 (244.28)	510.48 (227.65)
Area (km^2^)	0.59 (0.788)	5.27 (12.19)	27.84 (39.15)	26.81 (32.79)
Persons Aged 0–15	354.71 (127.08)	421.34 (167.73)	477.07 (201.51)	469.38 (168.12)
Persons Aged 16–39	619.93 (255.23)	659.02 (267.42)	679.09 (262.81)	646.26 (208.13)
Persons Aged 40–64	558.87 (139.07)	644.75 (192.15)	728.76 (210.96)	709.65 (200.41
Persons Aged 65+	303.83 (104.89)	337.75 (124.20)	359.30 (133.89)	326.30 (118.56)
Protestant or Other Christian	46.63 (30.68)	47.57 (28.87)	49.30 (28.83)	52.05 (28.52)

### Regression results

The results of the regression analyses are presented in Tables 3 and 4. Model 1 shows the results of the multinomial regression, focusing on factors related to the proportion of the area level population living within 1.5 km of a GP practice – grouped into full coverage, high coverage, low coverage, and no coverage. This model shows that, compared to areas with no coverage, areas with high and full coverage are more health deprived. The results also show that areas with full coverage have a lower number of people aged 0–15 when compared with areas with no coverage. Conversely, areas with a higher number of people aged over 65 have higher levels of coverage. In earlier models, other age bands were included, but were removed in the final models due to high levels of multicolinearity, probably occurring because of children and their parents cohabiting. The results also show a significant negative relationship between the proportion of people who report being protestant, and the level of GP coverage. Smaller areas also have higher levels of GP coverage.

**Table 3:** Model 1 shows the multinomial regression including Age, area, religion, and health deprivation influences on coverage. Reference category is no coverage. Model two shows the relationships with average travel distance

	Model 1			Model 2
	Low coverage	High coverage	Full coverage	Average distance
Health Deprivation and Disability (reverse coded)	.000 (.000)	.001** (.000)	.002*** (.001)	−1.143*** (.306)
Population aged 0–15	.000 (.001)	−.001 (.001)	−.002*** (.001)	1.387** (.437)
Population aged 65+	.003*** (.001)	.004*** (.001)	.006*** (.001)	−.855 (.630)
Protestant	−.010** (.004)	−.012*** (.004)	−.012** (.005)	−.346 (2.756)
Area (km^2^)	−.004 (.003)	−.051*** (.007)	−1.272*** (.224)	45.741*** (2.653)
Intercept	−.079 (.472)	.002*** (.476)	.448*** (.567)	2165.628*** (337.347)
N	890			
Log likelihood	2010.943***			
	Nagelkerke R^2^: .412			R^2^:.352

*p < 0.10; **p < 0.05; ***p < 0.01. Unstandardised coefficients with standard errors in parentheses

**Table 4:** Model 3 shows the multinomial regression including Age, area, religion, and multiple deprivation influences on coverage. Reference category is no coverage. Model 4 shows the relationships with average travel distance

	Model 3			Model 4
	Low Coverage	High Coverage	Full Coverage	Average Distance
Multiple Deprivation (reverse coded)	.000 (.000)	.001** (.000)	.002*** (.000)	−0.616** (0.296)
Population aged 0–15	.000 (.001)	−.001 (.001)	−.002** (.001)	1.329*** (0.440)
Population aged 65+	.003 (.001)	.004*** (.001)	.006*** (.001)	−0.747 (0.637)
Protestant	−.009** (.004)	−.013*** (.004)	−.012** (.005)	1.351 (2.736)
Area (km^2^)	−.003 (.003)	−.055*** (.007)	−1.312*** (.225)	48.574*** (2.567)
Intercept	−.127 (.465)	.053** (.466)	.528 (.550)	1791.466*** (328.601)
N	890			
Log likelihood	2011.011***			
	Nagelkerke R^2^:.412			R^2^:.345

*p < 0.10; **p < 0.05; ***p < 0.01. Unstandardised coefficients with standard errors in parentheses

Model 2 (Table 3) focuses on the relationships with the average network distance that must be travelled by the population to reach their nearest GP practice. This model shows that areas with higher levels of health deprivation have lower travel distances to their nearest GP practice. There is a significant positive relationship between the number of people aged 0–15 and the average distance to the nearest GP practice. However, the number of people aged over 65 is not significant, nor is religion. A significant positive relationship is found between the size of the area and the distance that must be travelled to reach the nearest GP practice.

Model 3 focuses on the proportion of the population of each area within 1.5 km of a GP practice, but unlike model 1, it focuses on multiple deprivation rather than specifically focusing on health deprivation. Compared to low coverage areas, high and full coverage areas tend to have higher levels of deprivation. The number of people aged over 65 is significantly positively related to higher levels of coverage, whereas the number of people aged under 15 is significantly negatively related to full coverage. There is a significant negative relationship between the proportion of the area level population reporting as Protestant and Other Christian and higher levels of coverage. The results also show that smaller areas have higher levels of coverage.

Model 4 focuses on the relationships with average travel distance, including multiple deprivation rather than health deprivation. The results of this model show a significant negative relationship between deprivation and travel distance. The proportion of the population aged under 15 is significantly positively related to the average travel distance, but no evidence is found for a relationship between the proportion of the population aged over 65 and travel distance. The proportion of people who report as Protestant and Other Christian is not found to be significant. Evidence is also found for a positive relationship between the size of the area and the average travel distance.

### Results of the sensitivity analysis

Sensitivity analysis was carried out by constructing a series of dependent variables based on different network distances of 0.75 km, 1 km, 1.25 km, 1.75 km and 2 km, and comparing the results of these models with the focal 1.5 km model. Although the overall patterns were similar, this analysis revealed some differences in the models when different distances are used. This section reviews the patterns in the differences that were found. For the purposes of this section, significance is considered at the 5% level. Although, the population aged 0–15 was only significant in the full coverage models presented previously, significant positive relationships were also found in all sensitivity models at the high coverage level, and at the low coverage level in the two models based on a distance of 1.25 km. Health deprivation and MDM were found to be significant in the 0.75 km and 1 km models, but MDM was not significant in the model based on 1 km distance when considering full coverage. The SOA area was also found to be significant in the health deprivation models when considering distances of 0.75 km and 1 km. Some differences also emerged when focusing on religion. In contrast to models 1 and 3, the number of people reporting protestant religion was found not to be significant in the models for full coverage based on 0.75 km and 1 km, nor in the low coverage models based on distances of 1.75 km and 2 km. Moreover, the overall variance explained by the models increased as the distance used to calculate the dependent variable was increased. Across all models the lowest Nagelkerke R-squared was 0.266 in the model based on a distance of 0.75 km, including health deprivation. The highest R-squared was 0.513, which resulted in both models based on the 2 km distance.

## Discussion

This study focuses on the relationships between primary care accessibility and population characteristics in Northern Ireland. The results provide insight into the relationships between the population characteristics and the location of GP practices, adding to our understanding of the populations in which GP practices are located. The healthcare planning assumption underpinning the study is that GP practices should be located closest to areas with the greatest need, and therefore relationships are expected between population need and accessibility.

The results of the regression analysis support the proposition that the most deprived areas should have the highest levels of GP coverage. This is the case for both overall multiple deprivation and for health deprivation, even when controlling for area size, population age, and religion. This relationship exists when accessibility is measured using coverage and average distance. The only models where deprivation is not significant are those comparing no coverage with low levels of coverage. This finding suggests that GP practices are located in areas with highest levels of deprivation related need. The relationship between deprivation and facility location observed in this study is consistent with findings from the wider literature [10, 25]. This importance of this finding is further highlighted by the negative relationship between deprivation and population health discussed in the literature [46–50].

The population age is an important consideration in the location of healthcare facilities as older people tend to have increased mobility problems [51], and also suffer from increased health problems [52]. The findings presented here show that areas with older populations also have higher levels of coverage even when controlling for other factors such as deprivation, area size, and religion. However, no evidence is found for a relationship between the population aged 65 and over and average travel distance. Areas with more people aged under 15 are associated with higher average travel distance. Moreover, areas with full coverage have lower numbers of people in this age group. These findings suggests that there is not necessarily a trade-off between coverage in deprived areas and coverage in areas with an older population.

Smaller areas tend to have higher levels of coverage, which shows that GP’s are located closer to population centres, where they can serve more dense populations. Although this make sense conceptually, consideration also needs to be given to how the primary healthcare needs of more rural areas are met, whether this is through GP practices or alternative services. These findings are consistent with previous research showing substantially better access to GP practices in urban areas in England [10] and Ireland [53]. The results also show some evidence of differing access to GP practices by religion. Historically, religious divides have been prominent in Northern Ireland, with certain areas continuing to remain predominantly Protestant or Catholic.

Although the overall general trend is similar across the models, the sensitivity analysis does show some differences across models. These differences highlight the importance of considering different levels of coverage when examining accessibility. The population aged 0–15 may be more important for other distances than the focal distance presented here. Religion may be less important for other distances. One potential explanation for the differences in the levels of significance could be the movement of people from one category to another as the distance changes, therefore changing the number of people in each category. Healthcare planners may therefore want to consider multiple distances when examining GP practice coverage. Moreover, the larger the distance considered in the dependent variable the more variance the model explains.

Although this study contributes to the current body of literature on the geography of healthcare facilities, it is not without limitations. This study focuses specifically on coverage as one measure of accessibility, but does not consider which facilities people choose to attend. Although there is some evidence from other contexts to suggest people tend to choose one of the closest facilities [54], future studies could consider whether people choose to attend the facility that is closest to home. This study also considers two specific measures of accessibility, and there are other potential measures that future studies could incorporate to build on this research. Future research could consider the specific population needs and service profiles of the GP practices, as well as the impact of need and accessibility on utilisation. This research also focuses specifically on factors that may influence the need for primary care services, rather than focusing on the factors related to the utilisation of services. Future work could consider which factors drive need, accessibility, and utilisation of services, and whether these factors are the same. Consideration of other healthcare services could also provide additional insight for healthcare decision makers. Moreover, the measures considered here do not consider the ability of the healthcare provider to deliver the care. Future studies could therefore incorporate factors such as the number of GP’s and other staff working at the practice, the practice list size, specialist services, and opening hours matched to population need.

## Conclusions

This study focuses on the relationship between population characteristics and GP practice accessibility. The findings of the research show that GP practices are located in areas with higher levels of need, as measured by older populations and deprivation. This research contributes to the existing methodological and empirical literature through the examination of multiple variables which should influence the location decision making process. From a policy perspective, the findings indicate some of the factors that are associated with GP location. However, a significant proportion of the population live outside of the 1.5 km coverage area, which is consistent with the rural geography of Northern Ireland. Planners should consider how best to serve this population, for example through other means of care, such as pharmacies and district nursing services. Long travel distances in rural areas also highlight the need for either private or public transportation to reach primary care services. From a methodological perspective, this study also highlights the importance of considering multiple measures of coverage and accessibility when considering the location of GP practices.

The incorporation of the data and analysis presented here into a planning tool could assist with the location decision-making process, as well as the wider provision of healthcare services. This is particularly important in the current Northern Ireland healthcare system, where the role of GP’s and pharmacies are evolving to meet healthcare needs. For example, current GP practice developments include practice based pharmacists, advance nurse practitioners, telephone triage, and online services [28]. The implementation of these innovations should be driven by evidence, and informed by the local population characteristics. Moreover, this evidence should be considered alongside alternative services, such as the pharmacy minor ailments scheme [27]. GP’s could also use this information to plan their own staffing requirements based on population need. This is likely to become a more prominent concern in Northern Ireland which has an ageing GP workforce [55]. In addition, the analysis of accessibility and population characteristics could be used retrospectively to evaluate the performance of past locational decisions. If locational decision making processes are functioning effectively high levels of correlation would be expected between the factors which are driving healthcare need and the location of the facilities.

## Abbreviations

GP: General practitioner
Km: Kilometre
km^2^: Square kilometres
Max: Maximum
MDM: Multiple deprivation measure
Min: Minimum
SD: Standard deviation
SOA: Super output area
